# P-257. Effectiveness of Pulsed-Xenon Ultraviolet Disinfection during Terminal Cleaning of Patient Rooms in the Intensive Care Unit on the Sustained Containment of Drug-resistant *Acinetobacter baumannii*

**DOI:** 10.1093/ofid/ofae631.461

**Published:** 2025-01-29

**Authors:** Keita Morikane, Jun Yakuwa, Masaki Nakane

**Affiliations:** Yamagata University Hospital, Yamagata, Yamagata, Japan; Yamagata University Hospital, Yamagata, Yamagata, Japan; Yamagata University Hospital, Yamagata, Yamagata, Japan

## Abstract

**Background:**

Environmental disinfection by ultraviolet (UV) has been shown to be effective in controlling transmission of multidrug-resistant organisms, and introduced into daily practices in addition to manual terminal cleaning in some hospitals in the US. However, its effectiveness outside the US healthcare setting is not well investigated. Moreover, its effectiveness to control gram-negative bacteria has not been well described.

**Methods:**

This study was conducted in the intensive care unit (ICU) of Yamagata University Hospital, a 637-bed tertiary referral hospital. Sporadic transmission of two drug-resistant *Acinetobacter baumannii* (2DRA) began in late 2013. Despite intensified hand hygiene promotion and manual terminal cleaning, transmission of 2DRA continued. In January 2018, we added pulsed-xenon ultraviolet (PX-UV) disinfection for the terminal cleaning after every patient discharge. The study periods were defined as follows: the baseline period (August 2016 to January 2018, manual cleaning only) and the intervention period (February 2018 to December 2023, addition of PX-UV). All patients admitted in the ICU were regularly screened for 2DRA for the detection of possible acquisition of 2DRA throughout the study periods.

**Results:**

The incidence of acquisition of 2DRA in the ICU significantly declined (4.45 per 1,000 patient days in the baseline period to 1.09 in the intervention period, relative risk (RR): 0.26, 95% confidence interval: 0.12-0.51). Cases in the intervention period were sporadic, and no cluster was found. In contrast, in non-ICU wards, where PX-UV disinfection was performed only after the discharge of patients known to be colonized or infected with 2DRA or other multidrug-resistant pathogens, possible transmission of 2DRA was observed several times.

**Conclusion:**

Adding PX-UV after manual terminal cleaning was effective in controlling acquisition of 2DRA in the ICU patients. The effectiveness of PX-UV in controlling gram-negative MDROs in the non-US healthcare settings is suggested. In contrast, there was room for improvement in controlling of 2DRA in non-intervention wards.

**Disclosures:**

**All Authors**: No reported disclosures

